# Construction Notes

**Published:** 1903-11-21

**Authors:** 


### CONSTRUCTION NOTES.

The new Levy Ward at Charing Cross Hospital, founded by Miss Matilda Levy, was opened last week. The ward contains twelve beds and is for the reception of female Jewish patients principally. It is provided with a special kitchen for the use of Jewish patients.

The new Hospital for Women and Children, which was opened recently by the Viscountess Mountgarret, consists of a new administrative department (some of the domestic offices remaining in the old building), the ward pavilion of two storeys, operation department, mortuary, and a maternity department. Hitherto there has been no special hospital accommodation for lying-in cases in Leeds. This new department is situated on the third floor of the administrative building. It consists of four wards, and is entirely self-contained. The architects are Messrs. Chorley and Common, of Leeds, assisted by Mr. Alexander Graham.

A new isolation hospital has been opened for the Urban District Council of Epsom. It consists of three blocks for scarlet fever, typhoid, and diphtheria, and also administration, laundry, and disinfecting blocks. The architect is Mr. E. R. Capon, surveyor to the Board.

The Bradford Union have opened a sanatorium for consumptives at Bardon Moor, on a site 800 feet above the sea. The patients are located at present in a long one-storeyed building with the exception of the central portion, over which there is a room for nurses. It contains four single bedrooms, four two-bedrooms, one for four, and one for five patients. It is intended to build another wing with the same accommodation later. The structure is of wood roofed with tiles. The administration block is at the rear of the patients' pavilion. Shelters will be built in the grounds. The estimated cost was about £10,000, or about £192 per bed. The plans were prepared by Mr. F. Holland, engineer and architect to the Bradford Guardians.

